# Association between triglyceride-glucose index and acute kidney injury in patients with acute myocardial infarction based on medical information mart for intensive care database: A cross-sectional study

**DOI:** 10.5937/jomb0-45219

**Published:** 2024-01-25

**Authors:** Zihan Jin, Lu Xiao, Xinyi Xu, Changhong Miao, Yi Liu

**Affiliations:** 1 Tianjin University of Traditional Chinese Medicine, Tianjin, China; 2 University of Traditional Chinese Medicine, First Teaching Hospital of Tianjin, Tianjin, China; 3 National Clinical Research Center for Chinese Medicine Acupuncture and Moxibustion, Tianjin, China

**Keywords:** acute kidney injury (AKI), triglyceride glucose index (TyG), acute myocardial infarction (AMI), Medical Information Mart for Intensive Care (MIMIC), akutna povreda bubrega (AKI), indeks triglicerida i glukoze (TiG), akutni infarkt miokarda (AMI), Medicinski informacioni centar za intenzivnu negu (MIMIC)

## Abstract

**Background:**

The relationship between triglyceride glucose (TyG) index and the incidence of acute kidney injury (AKI) in patients with acute myocardial infarction (AMI) is unclear. This study aims to explore the relationship between the two.

**Methods:**

Participants were enrolled from Medical Information Mart for Intensive Care IV (MIMICIV) and grouping of subjects based on the quartile interval of the TyG index. With the presence of AKI as the main outcome, a logistic regression model was constructed. The correlation of the TyG index with the results obtained was examined by using a restricted cubic spline (RCS) model.

## Abstract (sr)

**Uvod:**

Veza između indeksa triglicerida glukoze (TiG) i incidence akutne povrede bubrega (AKI) kod pacijenata sa akutnim infarktom miokarda (AMI) nije jasna. Ova studija ima za cilj da istraži odnos između njih.

**Metode:**

Učesnici su upisani iz Medical Information Mart for Intensive Care IV (MIMICIV) i grupisanje ispitanika na osnovu kvartilnog intervala TiG indeksa. Uz prisustvo AKI kao glavnog ishoda, konstruisan je model logističke regresije. Korelacija TiG indeksa sa dobijenim rezultatima ispitana je korišćenjem modela ograničenog kubnog splajna (RCS).

**Rezultati:**

Među 1.101 učesnikom AMI, 64,7% su bili muški pacijenti, a ukupna stopa incidencije AKI je bila 37,1%. Rezultati multivarijantne logističke regresione analize (LRA) otkrili su nezavisnu korelaciju TiG indeksa sa povećanim rizikom od AKI (odnos šanse (OR) = 2,23; interval poverenja (CI) = 1,59 do 3,17; P < 0,001). K4 (9,5-12,208) u kvartilu TiG indeksa je nezavisno korelirao sa povećanim rizikom od AKI (OR = 2,99, 95% CI = 1,64 do 5,46, P < 0,001). RCS model je pokazao da se rizik od AKI linearno povećavao sa povećanjem TiG indeksa (P=0,313).

**Zaključak:**

Kod pacijenata sa AMI, incidencija TiG i AKI je usko povezana. Ipak, potrebne su dalje studije kako bi se potvrdio nalaz.

## Introduction

Acute kidney injury (AKI) has been recognized as one of the common complications of myocardial infarction. Previous studies have shown that the incidence of AKI in patients with acute myocardial infarction (AMI) is 16%-20.4% [1] [2] [3]. Indeed, AKI causes approximately 17 million hospitalizations per year in the United States, costing the health care system more than $10 billion each year [4].

The mortality of AMI patients with AKI is high. Previous studies have demonstrated that the shortterm and long-term mortalities of AMI patients with AKI were 3.99% and 2.43%, respectively [5]. AKI is characterized by a sudden yet reversible decrease in estimated glomerular filtration rate (eGFR) in the early stages of AKI [6]. Therefore, an indicator is needed for early diagnosis of AKI to enable early clinical intervention.

The understanding of AKI has been greatly improved over the past decades, while its early diagnosis remains challenging. Kidney function index and serum creatinine exhibit poor performance for early diagnosis of AKI [7], and effective and reliable markers are urgently needed.

The existing research shows that TyG index is closely related to the occurrence of AKI [8]. However, the specific correlation of the TyG index with AKI in AMI patients remains unclear. Hence, the correlation of the TyG index with AKI in AMI patients was investigated in this study.

## Materials and methods

### Target population

**Figure 1:**
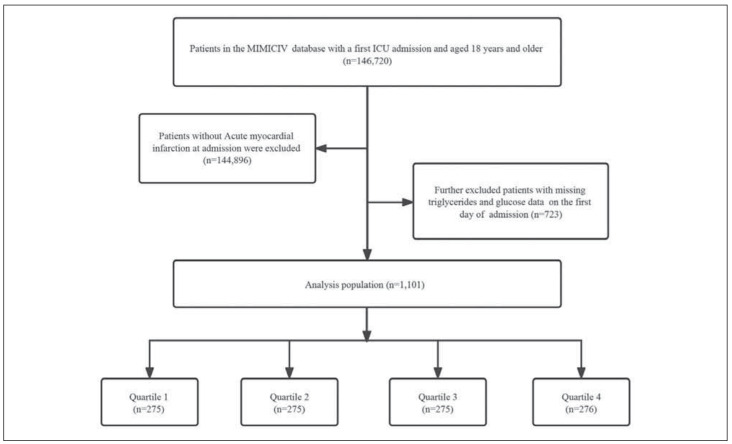
Flowchart of the study cohort.

The data were obtained from the online available Medical Information Mart for Intensive Care IV (MIMICIV) database (certificate number = 53477982) [9]. The need for informed consent was waived. Patients whose diagnosis of AMI was not the first in the diagnostic sequence were excluded. Subsequently, we further excluded patients with missing triglyceride (TG) and glucose data on the first day of hospital admission. In this study, 1,101 patients were enrolled and grouped on the basis of the TyG index quartile on the initial day of intensive care unit admission (Figure 1).

### Data collection

The Structured Query Language (SELECT) of Navicat Premium (Version 16) was employed for extraction of baseline characteristics (e.g., gender, age, laboratory indicators, and comorbid conditions). The TyG index was calculated by using the following equation: ln (fasting total cholesterol (mmol/L) × fasting blood glucose (mmol/L)/2). Additionally, cerebral infarction, respiratory failure, diabetes, dyslipidemia, atrial fibrillation, hypertension, heart failure, chronic kidney disease, AKI, and AMI were determined by using ICD-9 or-10 codes. In MIMICIV, missing data are common, but missing values of all screening variables are less than 5%. Therefore, no interpolation was executed in this study.

### Key outcomes and clinical definitions

In this study, the key outcome was the occurrence of AKI. According to the Kidney Disease: Improving Global Outcomes (KDIGO), AKI is defined as the case where baseline serum level of creatinine (sCr) 0.016 mmol/L for 48 h [10].

### Statistical analysis

As indicated, the distributions of baseline data of different groups were different. Disaggregated and continuous data were expressed as numbers (percentage) and median (interquartile range) or mean ± standard deviation (SD), respectively.

Continuous variables were investigated by using rank-sum tests or analysis of variance (ANOVA). The chi-square test or Fisher's exact test for categorical variables was used to compare the features of study subjects in the outcome groups.

As lymphocyte, high-density lipoprotein (HDL), total cholesterol (TC), and neutrophil percentages of missing data were small (missing rate = 1.72%-3.45%), no interpolation was required in this study. The independent relation of the incidence of AKI with the TyG index was assessed by means of logistic regression analysis (LRA). The TyG index was input as a categorical variable (quartile) or a continuous variable (OR was calculated for each additional unit) and four models were employed for the regression analysis. Herein, Model 1 was kept untouched; Model 2 was adjusted according to age, gender, and race; Model 3 was adjusted based on Model 2, accompanied by HDL, hemoglobin, lymphocytes and neutrophils, platelet count, potassium, red blood cells, serum creatinine (sCr), sodium, TC, urea nitrogen, white blood cells, eGFR, and triglycerides; Model 4 was adjusted based on Model 3, accompanied by atrial fibrillation, cerebral infarction, chronic kidney diseases, hyperlipidemia, hypertension and respiratory failure.

A restricted cubic spline model was employed to clarify the possible linear relation of the incidence of AKI with the TyG index. Specifically, the lower quartile of the TyG index was used as the reference group, and the covariable (Model 4) was adjusted accordingly. Additionally, the P-value of the trend was calculated with the quartile level as the order variable.

Furthermore, the analysis was stratified according to gender, heart failure, cerebral infarction, and hypertension to determine the robustness of the TyG index in predicting the risk of AKI. The interaction of the variables for stratification and the TyG index was investigated by using the likelihood ratio test. A twotail test was executed. R package 1.7.1 was used for all analyses [11] and P<0.05 denoted statistical significance in this study.

## Results

In this study, 1,101 patients were enrolled as the participants. Herein, the average age of the 712 (64.7%) male participants was 64.5±12.9 years old. The average TyG index of the participants was 9.1±0.8 and their incidence of AKI was 37.1%. According to the 2018 AHA/ACC Cholesterol Guidelines and the blood glucose standards published by WHO, the normal value range of TyG index is 8.95 to 10.13.

### Baseline features

Table 1 lists the quartiles of the TyG index and the baseline characteristics of the participants. Herein, the participants were divided into different quartiles (Q1:6.755-8.538) (Q2:8.54-8.971) (Q3:8.972-9.498) (Q4:9.5-12.208) according to the TyG index at admission, and their average TyG indexes were 8.2 ± 0.3, 8.8 ± 0.1, 9.2 ± 0.2 and 10.1 ± 0.5, respectively. As indicated, the prevalence rates of chronic kidney disease, diabetes, heart failure, and respiratory failure, higher levels of blood glucose, triglyceride, and TC were proportional to the TyG index, and the level of HDL was inversely proportional to TyG index. Additionally, the incidence of AKI gradually increased as the TyG index increased (29.8% vs. 32.7% vs. 34.9% vs. 51.1%, P<0.001).

**Table 1:** Baseline characteristics of critical patients with acute myocardial infarction grouped according to TyG index quartiles. ^a^ TyG index quartiles: Q1 (6.755–8.538), Q2 (8.54–8.971), Q3 (8.972–9.498), Q4 (9.5–12.208)<br>Abbreviations: HDL, high-density lipoprotein; TC, total cholesterol; eGFR, estimated glomerular filtration rate; TyG, triglyceride glucose index

Categories	Total (n = 1101)	Q1 (n = 275)	Q2 (n = 275)	Q3 (n = 275)	Q4 (n = 276)	P value
Gender						0.354
Female	389 (35.3)	87 (31.6)	105 (38.2)	94 (34.2)	103 (37.3)	
Male	712 (64.7)	188 (68.4)	170 (61.8)	181 (65.8)	173 (62.7)	
Age	64.5 ± 12.9	65.5 ± 13.0	66.3 ± 13.5	63.8 ± 12.5	62.2 ± 12.3	< 0.001
Race, n (%)						0.01
Asia	41 ( 3.7)	8 (2.9)	10 (3.6)	12 (4.4)	11 (4)	
Black	140 (12.7)	50 (18.2)	34 (12.4)	22 (8)	34 (12.3)	
Latino	59 ( 5.4)	11 (4)	11 (4)	11 (4)	26 (9.4)	
White	763 (69.3)	181 (65.8)	198 (72)	203 (73.8)	181 (65.6)	
Other	98 ( 8.9)	25 (9.1)	22 (8)	27 (9.8)	24 (8.7)	
Laboratory tests						
Glucose, mmol/L	6.4 (5.4, 8.8)	5.4 (4.9, 6.1)	6.1 (5.3, 7.0)	6.9 (5.7, 8.4)	11.2 (7.5, 15.5)	< 0.001
Triglycerides, mmol/L	7.1 (5.0, 10.3)	4.1 (3.3, 5.1)	6.4 (5.5, 7.5)	8.8 (7.2, 10.6)	13.1 (9.2, 19.2)	< 0.001
HDL, mmol/L	2.6 ± 0.9	3.1 ± 1.0	2.8 ± 0.8	2.4 ± 0.7	2.2 ± 0.7	< 0.001
Hemoglobin, g/L×10	13.3 ± 2.1	13.3 ± 1.8	13.2 ± 2.1	13.4 ± 2.0	13.3 ± 2.2	0.749
Lymphocytes, %	21.9 ± 10.9	22.2 ± 10.4	21.7 ± 11.1	21.8 ± 11.1	22.1 ± 11.0	0.958
Neutrophils, %	68.5 ± 12.7	68.0 ± 11.8	68.6 ± 13.0	68.9 ± 12.5	68.7 ± 13.3	0.890
Platelet count, ×10^9^/L	242.1 ± 79.9	230.1 ± 71.2	245.9 ± 86.5	243.0 ± 81.7	249.5 ± 78.5	0.025
Potassium, mEq/L	4.3 ± 0.7	4.3 ± 0.7	4.3 ± 0.6	4.2 ± 0.6	4.4 ± 0.7	0.055
Red blood cells, ×10^12^/L	4.4 ± 0.7	4.4 ± 0.6	4.4 ± 0.7	4.5 ± 0.7	4.5 ± 0.7	0.585
Serum creatinine,mmol/L	0.1 (0.0, 0.1)	0.1 (0.0, 0.1)	0.1 (0.0, 0.1)	0.1 (0.0, 0.1)	0.1 (0.0, 0.1)	0.052
Sodium, mEq/L	138.8 ± 3.5	139.3 ± 3.7	139.3 ± 3.5	139.1 ± 3.4	137.8 ± 3.3	< 0.001
TC, momol/L	9.8 ± 2.8	9.2 ± 2.5	9.6 ± 2.6	9.7 ± 2.8	10.7 ± 3.3	< 0.001
Urea nitrogen, mmol/L	1.0 (0.8, 1.3)	0.9 (0.7, 1.3)	1.0 (0.8, 1.4)	0.9 (0.8, 1.3)	1.1 (0.8, 1.4)	0.040
White blood cells, ×10^9^/L	8.4 (6.7, 10.7)	7.8 (6.3, 9.9)	8.3 (6.6, 10.8)	8.9 (7.1, 11.3)	8.7 (7.0, 11.0)	< 0.001
eGFR, mL/(min*1.73m^2^)	70.9 ± 27.6	73.4 ± 26.6	71.9 ± 28.8	70.5 ± 27.0	67.8 ± 27.8	0.107
TyG index	9.1 ± 0.8	8.2 ± 0.3	8.8 ± 0.1	9.2 ± 0.2	10.1 ± 0.5	< 0.001
Comorbidities, n (%)						
Atrial fibrillation	68 ( 6.2)	24 (8.7)	15 (5.5)	12 (4.4)	17 (6.2)	0.18
Cerebral infarction	81 ( 7.4)	18 (6.5)	21 (7.6)	13 (4.7)	29 (10.5)	0.069
Chronic kidney disease	160 (14.5)	24 (8.7)	39 (14.2)	32 (11.6)	65 (23.6)	< 0.001
Diabetes						< 0.001
NO	761 (69.1)	229 (83.3)	219 (79.6)	200 (72.7)	113 (40.9)	
I	18 ( 1.6)	1 (0.4)	2 (0.7)	6 (2.2)	9 (3.3)	
II	312 (28.3)	43 (15.6)	53 (19.3)	68 (24.7)	148 (53.6)	
Undetermined	10 ( 0.9)	2 (0.7)	1 (0.4)	1 (0.4)	6 (2.2)	
Heart failure	514 (46.7)	108 (39.3)	117 (42.5)	131 (47.6)	158 (57.2)	< 0.001
Hyperlipidemia	165 (15.0)	36 (13.1)	39 (14.2)	40 (14.5)	50 (18.1)	0.38
Hypertension	537 (48.8)	136 (49.5)	131 (47.6)	131 (47.6)	139 (50.4)	0.893
Respiratory failure	219 (19.9)	33 (12)	57 (20.7)	50 (18.2)	79 (28.6)	< 0.001
Events, n (%)						
Acute kidney failure	409 (37.1)	82 (29.8)	90 (32.7)	96 (34.9)	141 (51.1)	< 0.001a

### Correlation of TyG index with AKI

According to the results of multivariate analysis, the TyG index showed a significant correlation with the incidence of AKI (Table 2). Specifically, the incidence of AKI increased by 0.71 times for every 1 unit increase in the Model 3 compared with the Model 1 (P<0.001). Compared to the lowest-TyG subgroup (Q1), the incidence of AKI in the highest-TyG subgroup (Q4) increased by 0.53 times (P<0.001) after quartile stratification of TyG index.

**Table 2:** Multivariate logistic regression analyses of TyG index and incidence of acute kidney failure. Model 1: Not adjusted.<br>Model 2: Adjusted for age, sex, and race.<br>Model 3: Adjusted as for Model 2 plus high density lipoprotein, hemoglobin, lymphocytes, neutrophils, platelet count, potassium, red blood cells, serum creatinine, sodium, total cholesterol, urea nitrogen, white blood cells, estimated glomerular filtration rate, and triglycerides.<br>Model 4: Adjusted as for Model 3 plus atrial fibrillation, cerebral infarction, chronic kidney disease, hyperlipidemia, hypertension, and respiratory failure.

Categories	Model 1		Model 2		Model 3		Model 4	
	OR (95%CI)	P value	OR (95%CI)	P value	OR (95%CI)	P value	OR (95%CI)	P value
TyG	1.52 (1.29~1.79)	<0.001	1.77 (1.48~2.1)	<0.001	2.39 (1.71~3.34)	<0.001	2.23 (1.57~3.17)	<0.001
Quartile								
Q1(n=275)	1(Ref)		1(Ref)		1(Ref)		1(Ref)	
Q2(n=275)	1.15 (0.8~1.64)	0.462	1.16 (0.8~1.69)	0.444	1.2 (0.77~1.86)	0.427	1.11 (0.7~1.76)	0.667
Q3(n=275)	1.26 (0.88~1.81)	0.202	1.49 (1.03~2.18)	0.036	1.53 (0.96~2.45)	0.074	1.57 (0.96~2.56)	0.071
Q4(n=276)	2.46 (1.73~3.49)	<0.001	3.04 (2.1~4.41)	<0.001	3.47 (1.97~6.12)	<0.001	2.99 (1.64~5.46)	<0.001
P for trend		<0.001		<0.001		<0.001		

### Restricted cubic spline regression model

**Figure 2:**
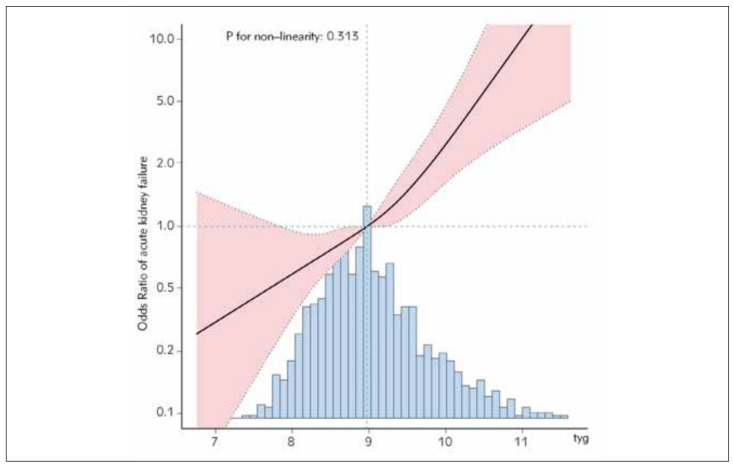
Linear dose-response relationship between triglyceride-glucose index and incidence of acute kidney failure<br>Adjustment factors included sex, age, race, high density lipoprotein, hemoglobin, lymphocytes, neutrophils, platelet count, potassium, red blood cells, serum creatinine, sodium, total cholesterol, urea nitrogen, white blood cells, atrial fibrillation, cerebral infarction, chronic kidney disease, hyperlipidemia, hypertension, respiratory failure, estimated glomerular filtration rate, triglycerides. The black line and pink line represent the estimated values and their corresponding 95% confidence intervals, respectively.

The restricted cubic spline regression model was employed for analysis and the following results were obtained. As indicated, the risk of AKI was proportional to the TyG index (P=0.313) (Figure 2).

### Stratified analysis of incidence of AKI by the TyG index

**Figure 3:**
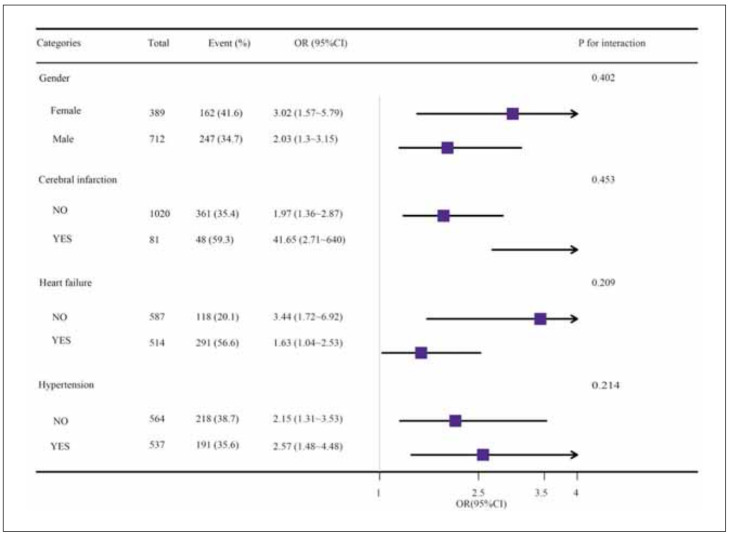
Subgroup analysis of the TyG index and acute renal failure.

After further assessment for different subgroups (e.g., gender, cerebral infarction, heart failure, and hypertension), the TyG index remained robust as a risk predictor of the primary outcomes (Table 3) and no significant interaction of different subgroups was observed (P>0.05 in all cases) (Figure 3).

**Table 3:** Effect size of TyG index on incidence of acute kidney failure in each subgroup.

Categories	No. of participants	n/N (%)	OR (95%CI)	P value	P for interaction
Gender					0.402
Female	389.0	162 (41.6)	3.02 (1.57~5.79)	0.001	
Male	712.0	247 (34.7)	2.03 (1.3~3.15)	0.002	
Cerebral infarction					0.453
NO	1020.0	361 (35.4)	1.97 (1.36~2.87)	<0.001	
YES	81.0	48 (59.3)	41.65 (2.71~640)	0.007	
Heart failure					0.209
NO	587.0	118 (20.1)	3.44 (1.72~6.92)	0.001	
YES	514.0	291 (56.6)	1.63 (1.04~2.53)	0.031	
Hypertension					0.214
NO	564.0	218 (38.7)	2.15 (1.31~3.53)	0.002	
YES	537.0	191 (35.6)	2.57 (1.48~4.48)	0.001	

## Discussion

To the best of our knowledge, this study is the first report focusing on the correlation of the TyG index with the incidence of AMI patients with AKI. The key finding of the study was that the TyG index was proportional to the incidence of AKI, even after adjustment for potential confounding variables. Additionally, this study provides a facile yet effective biomarker for early diagnosis of AKI in patients with AMI.

Currently, AKI is usually diagnosed based on the plasma level of creatinine. However, an increase in creatinine levels can only be observed for a few days after the kidney injury [12]. According to a previous study, serum creatinine is not a reliable indicator of acute kidney injury, which can lead to underestimation of the number of patients who have acute kidney injury during the acute phase [13]. Therefore, the diagnostic efficiency of plasma level of creatinine in AKI is limited. Therefore, a novel indicator of the occurrence of AKI is urgently needed.

The TyG index is a clinical surrogate for insulin resistance and it is often used to predict cardiovascular risk. The TyG index has been demonstrated to have a correlation with the occurrence and development of kidney diseases. Indeed, elevated TyG index has a significant correlation with arteriosclerosis and tubular epithelial cell damage [14]. Shi et al. [15] reported a strong correlation of the TyG index with decreased EGFR. Liu et al. [16] found that the TyG index was proportional to urinary albumin-to-creatinine ratio (UACR) in adults. Proteinuria was particularly associated with high TyG index in RHF patients [17]. Li et al. found that the TyG index showed a positive correlation with the kidney injury in middle-aged and elderly people with WRF [18]. Qin et al. [8] found that high TyG levels were positively related to the incidence of contrast-induced acute kidney injury (CI-AKI) only in diabetic patients. However, the correlation of the TyG index with AKI remains poorly understood.

As demonstrated, hyperglycemia is an independent predictor of AKI [19], while the specific mechanism of hyperglycemia after myocardial infarction remains unclear. Previous studies have shown that high cortisol levels in patients with AMI can lead to stress hyperglycemia [20]. Excessive glucocorticoid can promote gluconeogenesis, inhibit insulin, reduce glucose tolerance and cause hyperglycemia. However, the association of AMI and high cortisol levels has not yet been confirmed. Additionally, insulin resistance in the liver may lead to acute hyperglycemia after myocardial infarction [21]. Stress hyperglycemia is a risk factor for AKI and a contributing factor for AMI-induced AKI [22].

Dyslipidemia plays an inportant role in progression of kidney diseases, abnormal blood lipids themselves stimulate inflammation, leading to the destruction of cellular activity and pathological changes in renal tissue [23]. Despite that the specific mechanism remains unclear, lipids may damage tubular cells, mesangial cells and blood vessels of the kidney. Moorhead et al. firstly put forward »Lipid Nephrotoxicity Hypothesis« [23]. In addition, current research has shown that AKI can lead to the accumulation of cholesterol and triglycerides in the kidney, this also demonstrates the relationship between the TyG index and the AKI [24] [25].

The TyG index can monitor both triglyceride and glucose, and it is a reliable proxy for IR. The TyG index is readily accessible as the triglyceride and glucose levels can be obtained by conventional measurements in a cost-effective way. Research has shown a close relationship between TYG and other indicators for predicting AKI. The Dong's study shows TyG index is an influential factor in serum 2-microglobulin and Cystatin C levels [26]. A study in an elderly hypertensive population showed that the TyG index was proportional to uric acid [27]. This could also prove that there is an intrinsic link between the TyG index and the occurrence of AKI. Therefore, the TyG index was employed to analyze the incidence of AMI patients with AKI.

In summary, the TyG index is an AKI predictor with high reliability and validity. Patients with AMI with higher TyG index have higher risk of suffering from AKI. This study allows rapid identification of high-risk patients, thereby facilitating early diagnosis and clinical management of AKI. Additionally, this study is the first report investigating the correlation of the TyG index with AMI patients with AKI.

### Research limitations

First, the causality cannot be demonstrated in this cross-sectional survey. Hence, further cohort studies are needed to verify the correlation of TyG index with AKI. Nevertheless, multifaceted and rigorous statistical methods were employed to make the results as valid and reliable as possible. Second, the data used in this study were collected from patients in the US, and the applicability of the results to patients in China remains to be verified. Nevertheless, patients of different races were involved to minimize the raceinduced bias.

## Conclusion

For AMI cases, there is a potential correlation between the incidence of AKI and the TyG index, and our research provides a predictive index for the incidence of AKI. Further researches are needed to clarify the causal relationship between TyG index and AMI complicated with AKI.

## Dodatak

### Funding

This work has been supported by Tianjin Municipal Education Commission (No. 2021KJ144).

### Ethical approval and consent to participate

This study was conducted in accordance with the guidelines of the Declaration of Helsinki. The data used in this study are available online (in the MIMICIV database) and the study was exempted from ethical approval statements and informed consent.

### Availability of data and materials

The datasets generated and analyzed in this study can be accessed upon reasonable request.

### Author contributions

ZHJ conducted data extraction and analysis, and drafted the paper. LX, XYX, CHM and YL reviewed and revised the main knowledge points of the manuscript. And the submission of the paper was approved by all authors.

### Conflict of interest statement

All the authors declare that they have no conflict of interest in this work.

### Authorship

Zihan Jin and Lu Xiao have contributed equally to this work and share first authorship.
